# Pediatric Ogilvie Syndrome Following Laparoscopic Appendectomy: A Case Report and Literature Review

**DOI:** 10.7759/cureus.107181

**Published:** 2026-04-16

**Authors:** Jennyvette Trinidad-Pineiro, Humberto Lugo, Priscilla Dávila, Hiram J Diaz-Cortes, Cristhian G Negron Rodriguez, Sandra Cuevas-Rodriguez

**Affiliations:** 1 Surgery, University of Puerto Rico, Medical Sciences Campus, San Juan, PRI; 2 Pediatric Surgery, University of Puerto Rico, Medical Sciences Campus, San Juan, PRI

**Keywords:** acute colonic pseudo-obstruction, bowel obstruction, colonic decompression, neostigmine, ogilvie syndrome, pediatric surgery, postoperative complication

## Abstract

Acute colonic pseudo-obstruction (ACPO), or Ogilvie syndrome, is a rare cause of massive colonic dilation in children, occurring without mechanical obstruction. Its subtle presentation often mimics ileus or bowel obstruction, which may delay diagnosis and increase the risk of ischemia or perforation. We report the case of a seven-year-old boy who developed ACPO two weeks after a laparoscopic appendectomy for perforated appendicitis. Imaging revealed significant colonic distention without a transition point. The patient improved with supportive care, including bowel rest, intravenous fluids, and serial imaging, avoiding pharmacologic or surgical intervention. This case underscores the importance of maintaining a high index of suspicion for ACPO in postoperative pediatric patients with abdominal distention. Timely diagnosis and a stepwise management approach are essential to prevent complications and reduce the need for invasive treatment. A brief review of the literature is included to highlight current diagnostic and therapeutic strategies in pediatric ACPO.

## Introduction

Ogilvie syndrome, or acute colonic pseudo-obstruction (ACPO), involves significant colonic dilation without any mechanical blockage [1]. First described by Sir William Ogilvie in 1948 in patients with retroperitoneal malignancy [2], ACPO is uncommon in children, with most published data centered on adults, especially those in critical condition or recovering from surgery [3]. In pediatric patients, ACPO typically occurs following operations, severe infections, electrolytic disturbances, or the use of opioids and anticholinergic drugs [4,5].

The exact cause of ACPO remains unclear. Hypothesized mechanisms include autonomic dysfunction, reduced blood flow to abdominal organs, metabolic issues, malignancy, and neurotropic medications [5-9]. Pediatric symptoms may be nonspecific and resemble ileus or mechanical obstruction, complicating timely diagnosis. As distention worsens, the risks of ischemia and perforation grow. Early detection and prompt management are essential.

Treatment may involve supportive care, pharmacologic stimulation, endoscopic decompression, or surgery [10]. This report presents a pediatric ACPO case following laparoscopic appendectomy, alongside a literature review emphasizing diagnostic and treatment considerations in children.

## Case presentation

A seven-year-old boy with a medical history of attention-deficit/hyperactivity disorder (ADHD) presented to the emergency department (ED) with a three-day history of right lower quadrant abdominal pain, decreased oral intake, non-bilious emesis, subjective fever, and diarrhea. Imaging and clinical evaluation confirmed a diagnosis of perforated appendicitis. The patient underwent a laparoscopic appendectomy with findings of perforated appendix and periappendiceal purulent fluid. The fluid was evacuated via suction, and careful inspection of the abdominal cavity did not reveal widespread contamination or additional pathologic findings. Histopathology revealed a perforated appendix with an associated fecalith. On postoperative day (POD) 5, two small intra-abdominal abscesses were identified and successfully managed with a 14-day course of intravenous piperacillin-tazobactam (80 mg/kg/dose). The remainder of the postoperative course was uneventful, and the patient was discharged in stable condition, at baseline health.

Three days following discharge, he returned with new symptoms of reduced oral intake, recurrent non-bilious vomiting, and watery diarrhea of one-day duration. Physical examination revealed a distended and tympanic abdomen without signs of peritonitis or surgical site infection. Vital signs were within normal parameters. Laboratory results demonstrated borderline leukocytosis (white blood cell count at 11,680/μL), thrombocytosis (platelet count at 544 ×10³/μL), normal hemoglobin (12.8 g/dL), a mildly elevated erythrocyte sedimentation rate (33 mm/h), and normal C-reactive protein (1.3 mg/L). Electrolytes, renal function, and inflammatory markers were within normal limits, and testing for *Clostridioides difficile* was negative.

**Figure 1:**
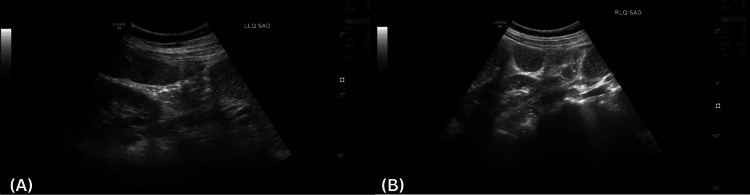
Sagittal sonographic view of the (A) right and (B) left lower abdominal quadrants demonstrating distended bowel loops measuring 3-3.5 cm in diameter.

**Figure 2:**
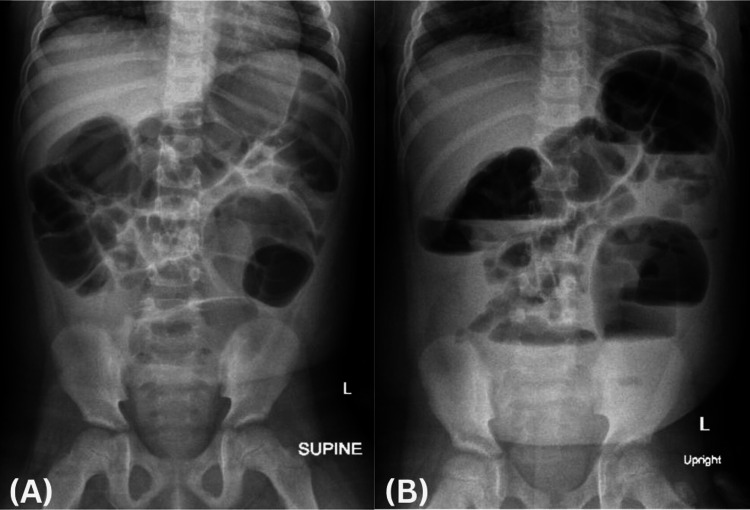
Initial radiographic evaluation at presentation to the emergency department. Anteroposterior abdominal radiographs obtained in the (A) supine and (B) upright positions demonstrate diffuse colonic distention with maximal diameter of 6 cm and no rectal air.

Initial diagnostic imaging included abdominal ultrasound and plain radiography. Sonographic evaluation revealed multiple distended bowel loops with diameters reaching up to 3.5 cm (Figure 1A, 1B). Abdominal radiographs demonstrated diffuse colonic gas accumulation with maximal dilation of 6 cm, notably without rectal air (Figure 2A, 2B). Although these findings raised initial concern for mechanical obstruction, the presence of diarrhea and colonic-predominant gas favored a diagnosis of ACPO.

The patient was admitted to the pediatric surgery service for supportive management, including intravenous fluid resuscitation, gastric acid suppression, bowel rest, serial laboratory assessments, and daily imaging. Over the subsequent 24 hours, clinical symptoms improved significantly, with resolution of vomiting and diarrhea, and reduction in abdominal distention. Despite clinical improvement, follow-up abdominal radiographs on admission days 2 and 3 showed persistent colonic dilation with air-fluid levels, suggestive of ongoing pseudo-obstruction.

**Figure 3:**
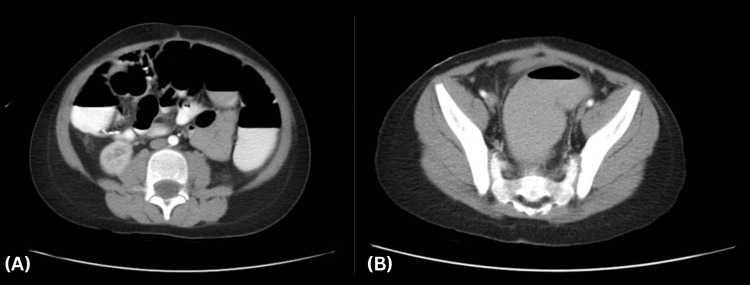
Third day of admission. Axial abdominal and pelvis CT scan demonstrates diffuse colonic dilation extending to the rectum without a transition point or evidence of mechanical obstruction, consistent with acute colonic pseudo-obstruction (ACPO). (A) Air-fluid levels are seen within the colon containing oral contrast. The cecum measures 5.0 cm, with surgical clips in place from the prior appendectomy. (B) Distended rectosigmoid colon with no transition point.

To further evaluate the persistent radiologic findings, an abdominal and pelvic computed tomography (CT) scan was performed (Figure 3A, 3B). CT imaging confirmed diffuse colonic dilation extending from the cecum (5.0 cm) through the transverse colon (4.7 cm) to the sigmoid colon (4.5 cm), without evidence of a transition point or mechanical obstruction. The previously documented intra-abdominal abscesses had resolved, and surgical clips from the prior appendectomy were visible in situ.

The patient remained hemodynamically stable and asymptomatic throughout his hospitalization, with progressive resolution of abdominal distention and no recurrence of symptoms. He was discharged in good condition on hospital day five, tolerating a regular diet, without abdominal distention on physical examination, and with return of normal bowel function. Appropriate outpatient follow-up was scheduled.

## Discussion

Ogilvie syndrome, or ACPO, is defined by marked colonic dilation without evidence of mechanical obstruction, a phenomenon that can present diagnostic and therapeutic challenges, especially in pediatric patients [7]. The clinical picture is often nonspecific, with progressive abdominal distention, pain, vomiting, constipation, and occasionally diarrhea, as seen in the patient presented here who initially exhibited distention, emesis, and watery diarrhea following recent abdominal surgery [3,11]. In children, such presentations may mimic more common entities like distal obstruction, Hirschsprung disease, or severe colitis, leading to delays in diagnosis and intervention [3,6]. Our case contributes to the limited pediatric literature, where reported ages range from 2 to 17 years and no clear sex predominance has been established [8,11-15]. The overlap in presentation emphasizes the need for heightened clinical suspicion and thorough exclusion of mechanical causes to reduce unnecessary interventions and avoid progression to complications [11,13].

Evaluation of ACPO relies on integrating clinical, laboratory, and imaging findings. Electrolyte imbalances such as hypokalemia, hypocalcemia, and hypomagnesemia have been implicated in the pathogenesis of ACPO and can also result from these deficits [16]. Diarrhea, as observed in our case, may exacerbate electrolyte loss, particularly potassium, further impairing colonic motility [17]. Although our patient did not demonstrate electrolyte abnormalities on presentation, other reports have identified hypokalemia as a frequent feature [9,13,18,19]. Hyperkalemia has also been described, albeit rarely [15]. These disturbances, particularly when combined with colonic distention, may lead to worsening bowel function and should be closely monitored and corrected. In our case, normal electrolyte levels throughout the admission simplified management, but serial laboratory surveillance remained essential. Additionally, secretory diarrhea may produce a non-anion gap metabolic acidosis secondary to bicarbonate loss, necessitating further biochemical assessment [20].

Radiologic assessment played a central role in diagnosing ACPO in our patient. Initial abdominal radiographs demonstrated colonic-predominant gas without rectal air, while ultrasound showed distended bowel loops - both typical features of pseudo-obstruction [21, 22]. While these findings may resemble mechanical obstruction, the clinical context - absence of transition point, presence of diarrhea, and lack of peritonitis - supported a diagnosis of ACPO. Advanced imaging with CT was used to confirm colonic dilation and rule out an obstructive lesion, demonstrating diffuse distention extending to the rectum with no identifiable transition point or ischemic signs [9,23]. Notably, the patient’s cecal diameter measured 5.0 cm, below the adult threshold of 9-12 cm typically associated with imminent perforation [22,24]. In pediatric cases, specific cutoff values have not been established, but reports describe colonic diameters ranging from 6 to 15 cm [9, 12], making close monitoring critical regardless of size.

Initial management of our patient was entirely conservative, aligning with recommended first-line strategies. He was treated with bowel rest, intravenous fluids, serial imaging, and correction of acid-base status, though no electrolyte abnormalities were present [22,23,25]. Rectal tube or pharmacologic decompression was not required, highlighting the potential for resolution with early, supportive measures in selected cases. In other pediatric and adult series, conservative treatment fails in up to 30% of cases, and recurrence can occur in up to one-third of patients [6,26]. Adult-based protocols suggest a 72-hour observation window before escalating treatment, though pediatric-specific timing remains to be defined [25]. Our patient improved rapidly, with symptom resolution by day two and radiologic improvement by day five, validating conservative management in early, stable presentations.

When conservative measures are insufficient, pharmacologic therapy - primarily neostigmine - becomes a second-line option. This acetylcholinesterase inhibitor stimulates colonic motility but must be used cautiously due to its cholinergic effects, including bradycardia and bronchospasm, which require continuous cardiac monitoring during administration [4,27,28]. Pediatric dosing is not standardized, but reported titrations up to 0.05 mg/kg have resulted in prompt decompression in several cases, with Gmora et al. (2002) [18] describing symptom resolution within hours. However, adverse events such as allergic reactions [14] and even cecal perforation [29] have been reported, emphasizing the importance of careful selection and monitoring. In our patient, neostigmine was not necessary, but its role should be considered in persistent or worsening cases without signs of perforation [12,18,30].

Erythromycin, a motilin receptor agonist, has also been explored as an adjunct or alternative therapy. While adult efficacy is inconsistent due to rapid tachyphylaxis [31,32], pediatric case reports show positive outcomes. Jiang et al. (2007) [11] and Gortani et al. (2019) [7] both reported successful resolution of ACPO in children using erythromycin within days. In a more complex approach, Chiacchio and Lowe (2021) [3] described a 10-year-old managed successfully with a combination of nasogastric and rectal decompression, erythromycin, and rectal tube placement. Although not used in our case, erythromycin remains a potential pharmacologic option, particularly where neostigmine is contraindicated or unavailable.

Endoscopic decompression is reserved for refractory cases or when pharmacologic therapy is not feasible. The Society of American Gastrointestinal and Endoscopic Surgeons (SAGES) guidelines recommend avoiding bowel preparation and limiting sedative use to preserve colonic motility, with the colonoscope ideally reaching at least the transverse colon [9]. While pediatric-specific thresholds for intervention remain undefined, adult studies report initial success rates up to 95%, with increased success after repeat procedures [6,33]. Our patient did not require endoscopic intervention, but persistent colonic distention on imaging did prompt close radiographic follow-up, a step critical to avoiding unnecessary procedures. Notably, Shukla et al. (2007) [9] documented successful resolution in a five-year-old using rectal tube decompression alone, suggesting that less invasive measures may be effective, particularly in pediatric patients with stable clinical status.

Surgical intervention is indicated in cases of perforation, peritonitis, or failure of nonoperative management [6,9,14]. Surgical options include decompressive stomas or resection with diversion, depending on the severity of contamination and bowel viability. In children with extensive contamination or unstable conditions, resection with protective ostomy is generally favored [34]. Our patient did not require surgical treatment, but the literature illustrates the potential severity of untreated or progressive ACPO. Garcia et al. (2018) [8] presented a 17-year-old with ACPO following spinal fusion who deteriorated to septic shock despite medical management. She underwent emergent laparotomy, revealing cecal perforation, and required ileocecal resection with ileostomy, emphasizing the importance of early recognition and intervention to prevent such outcomes.

## Conclusions

Pediatric acute colonic pseudo-obstruction is an uncommon but important postoperative complication that can easily be mistaken for other causes of intestinal dysfunction. This case illustrates how subtle symptoms such as abdominal distention, vomiting, or diarrhea can obscure the diagnosis and potentially delay timely intervention. Our patient’s favorable recovery with supportive measures alone highlights the value of maintaining a high index of suspicion and applying a careful, stepwise management strategy. Although standardized pediatric guidelines are lacking, individualized evaluation that integrates clinical status, laboratory monitoring, and imaging findings remains essential. Early recognition and tailored management can help prevent severe outcomes such as ischemia, perforation, and the need for surgical intervention.
